# Predictability of anthropomorphic measurements in implant selection for breast reconstruction: a retrospective cohort study

**DOI:** 10.1007/s00238-016-1261-z

**Published:** 2017-01-03

**Authors:** Egidio Riggio, Ilaria Ardoino, Caroline E. Richardson, Elia Biganzoli

**Affiliations:** 1 Unit of Plastic and Reconstructive Surgery, Fondazione IRCCS Istituto Nazionale dei Tumori, Via Venezian 1, 20133 Milan, Italy; 2 G.A. Maccacaro Unit of Medical Statistics, Biometry and Bioinformatics, Department of Clinical Science and Community Health, University of Milan, Milan, Italy; 3 Unit of Medical Statistics, Biometry and Bioinformatics, Fondazione IRCCS Istituto Nazionale dei Tumori, Milan, Italy

**Keywords:** Breast reconstruction, Anatomical implant, Mastectomy, Preoperative planning, Logistic regression, Decision-making, Breast implant sizes

## Abstract

**Background:**

Preoperative implant planning for breast reconstruction is often at risk of being changed perioperatively. This study examined which factors are associated with a change of implant selection.

**Methods:**

Women who had unilateral two-stage breast reconstruction between 2002 and 2007 were studied. Inclusion criteria were photographic evidence of preoperative skin markings indicating breast dimensions and a selected implant model. Multivariable logistic regression was used to identify variables associated with a changed selection.

**Results:**

Among the 496 women studied, 308 preoperative implant choices (62.1%) were changed during surgery. A change in plan was significantly associated with symmetrization surgery involving contralateral reduction mammaplasty (OR = 1.92; 95% CI, 1.12 to 3.29) and contralateral mastopexy (OR = 2.26; 95% CI, 1.29 to 3.96), but not with BMI. The required implant width changed more than 0.5 cm in 70 cases (14.1%) while height changed more than 0.5 cm in 215 cases (43.2%). The likelihood of a change was high for large preoperative widths (OR = 9.66 for 15.5 cm) and small preoperative heights (OR = 2.97 for 10.5 cm). At a mean follow-up of 16.6 months, patient satisfaction was good or average in 92.1% of cases and 5.9% of implants had been replaced with another model, indicating that the perioperative implant selection was usually appropriate.

**Conclusions:**

This study documents the frequency with which implant choices, despite accurate preoperative planning, are changed perioperatively as a result of relatively small differences in anthropomorphic measurements. Perioperative recalculation of breast dimensions may have an advantage in terms of patient reoperation rates. Changes in width were less frequent than changes in height and projection. Contralateral surgery, large width, and small height were the most influential factors.

Level of Evidence: Level IV, risk / prognostic study.

## Introduction

Before plastic surgery of the breast, the most accurate method to determine breast volume has long been direct volume and anthropomorphic measurement [1]. Three-dimensional body surface imaging by a 3D laser scanner is a new but expensive alternative to the classical methods of breast volume calculation such as anthropomorphic measurements, water displacement, thermoplastic castings, and nuclear magnetic resonance imaging [2–6]. Many studies continue to focus on volume assessment, but this is a poor method for implant selection now that anatomical expandable and silicone implants are available [7, 8].

Preoperative selection of breast implants on the basis of anthropometric measurements, in particular linear parameters (width, height, and projection), has been discussed in papers describing breast augmentation with the ultimate goal of achieving symmetry of shape and volume [9–12]. Implants for breast reconstruction are selected with the same approach [9–12], but in reconstructive surgery, it is difficult to avoid a certain degree of breast asymmetry. Preoperative planning for two-stage (expander and implant) reconstruction usually requires the selection of an anatomical device that matches the shape and size of the contralateral breast. Contralateral mammoplasty is considered when asymmetry of shape or size will potentially be excessive in the minds of the surgeon and patient. However, preoperative planning, even when accurate, cannot prevent errors in width, height, and projection measurements as it largely relies on the plastic surgeon’s experience and predictive capabilities. Moreover, preoperative measurements may not be sufficient for choosing the appropriate implant because in the perioperative phase, after removal of the expander, both the reobservation of anatomical features and the application of surgical refinements at the mastectomy site may require reevaluation of the anthropometric measurements for the optimal choice of implant. The aim of this retrospective study was to evaluate the relationship between the preoperative selection and actual choice of the implant in the perioperative setting in order to determine if there are predictable factors that could help standardize implant selection.

## Patients and methods

### Patients and data collection

We identified women who had undergone mastectomy and two-stage reconstruction surgery, with placement of a temporary expander before the final implant, at the Fondazione IRCCS–Istituto Nazionale dei Tumori, a national comprehensive cancer center, in the six-year period between 2002 and 2007. Patients were included if chest photographs with preoperative skin markings indicating the choice of implant were available. Patients who had undergone bilateral mastectomy were excluded.

**Fig. 1:**
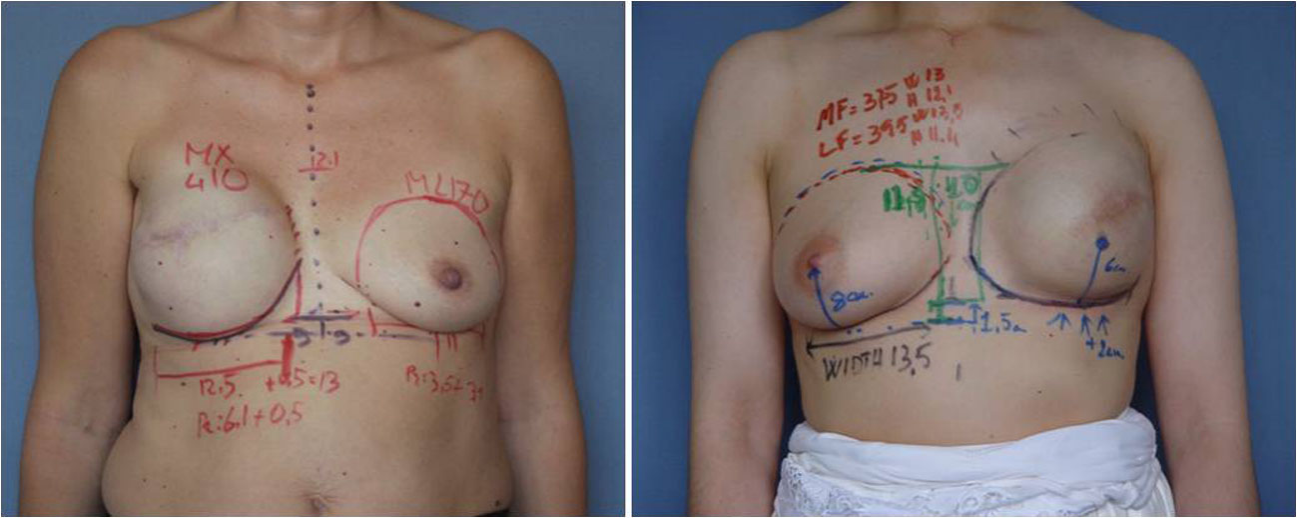
Preoperative photographs showing two complete drawings of the following parameters: width, height, and projection (cm); midsternal line; and inframammary fold level

From the clinical records, we collected data on each patient’s body mass index (BMI), model of implant used after expander removal, and contralateral breast-matching procedure (mastopexy, augmentation, reduction mammoplasty, or none). We retraced the preoperative plan from photographs (Fig. 1). In particular, we identified the implant that had been selected during the planning stage. We also recorded the measurements of breast implant width, height, and projection (all in centimeters, taken routinely with the patient standing) and noted if the sternal midline and inframammary fold (IMF) level had been marked. When all five of these markings were present and clearly visible, we considered the preoperative plan complete; if one or more markings were missing, we considered the preoperative plan partial. The measurements of breast dimensions taken from the photographs were verified against catalog values; for those cases in which these data were not retrievable from the photographs, we obtained missing information from the dimensions of the selected implant. Data for the weight (g) of each implant were obtained from the manufacturer’s catalog.

### Implant choice

In our unit, breast implants are chosen preoperatively by the lead surgeon, taking into consideration the following anthropomorphic variables: (a) breast width, height, and projection; (b) soft tissue thickness at the parasternal, upper, and lateral boundaries of the breast pocket; (c) asymmetry of the chest wall; (d) discrepancy between the predicted midpoint of the reconstructed breast and the contralateral nipple position; (e) IMF contour and level; (f) ptosis; and (g) possible planned contralateral surgery. Breast size and shape are subjectively assessed in relation to the expander volume, the indication for contralateral mammaplasty, and the patient’s expectations.

During the study period, the implant was initially chosen from the line of Allergan Inc. (Irvine, US) anatomical implants (Natrelle styles 410 and 510), which were fully stocked and readily available within the operating department complex (the 510 series has been available to us since 2003). The department also stocked the round implant styles 110 (moderate profile) and 120 (high profile), which the surgeon could opt for during the procedure.

### Implant preselection

The following anatomical landmarks are essential for the measurements: the midsternal line, suprasternal notch, nipple position, suprasternal nipple lines, midbreast longitudinal lines, inframammary transverse line, lateral boundaries, and inframammary contour of the non-affected breast.

A preliminary step in defining implant dimensions is observation of the footprint of the contralateral healthy breast. The contralateral IMF represents the fixed foundation for reconstruction of the missing breast. A flexible ruler is used to calculate the measurements for width and height, and a caliper calculates the measurements for projection and soft tissue thickness as obtained by the pinch test. All measurements are marked with the patient in the upright position.

The expected width of the reconstructed breast is derived from the definition of the contralateral breast’s horizontal boundaries. This width corresponds to the sum of the implant width and the thickness of the soft tissue overlying the expander both medially and laterally. The medial limit of the implant width should not be less than 1 cm from the midsternal line.

Correct height does not depend solely on the chest somatotype or the upper pole desired by the patient. Implant height is derived from the height of the contralateral breast, but we must also take into account possible post-mastectomy soft tissue loss exceeding the upper boundaries of the breast parenchyma. Other factors such as rebalancing the nipple-areola complex after a nipple-sparing mastectomy can modify the expected height of the reconstructed breast, especially when associated with augmentation of the contralateral breast.

The projection of the reconstructed breast is derived from the definition of the contralateral breast projection, which is calculated by pushing the central part downward and measuring the perpendicular projection of the areola from the chest wall. This projection corresponds to the addition of the implant projection to the thickness of the soft tissues overlying the central part of the expander.

### Surgical method

For the second stage of breast reconstruction, the preoperative planning and actual operation were performed by one of six consultant surgeons having good skill of breast surgery techniques (from 3 to 17 years of specific institutional practice) and following a standard approach for inscribing the preoperative marks. The practice in our unit is to perform contralateral surgery for symmetrization, if indicated, during this second stage, when the expander is replaced with the final implant. Preoperative planning did not include lipofilling. All operations were performed under general anesthesia with the patient positioned supine on the operating table, with the arms adducted and the elbows in moderate flexion. The position on the table was secured to allow movement through 90° into an upright sitting position; this crucial maneuver was always performed before making the final decision about which implant to use. The mastectomy incision was reopened, the expander was removed, and anterior capsulectomy was routinely performed. The IMF was recreated if necessary. The measurements of local tissue thickness were repeated and the implant choice was reviewed. The definitive implant was selected and inserted using standard aseptic methods.

### Follow-up

Follow-up data drawn from prospective clinical records were routinely recorded by the surgeon. In particular, we collected the following information: date of the last follow-up in order to calculate the duration in months, occurrence of complications, cosmetic outcome including shape and symmetry, patients’ satisfaction level, presence of capsular contracture graded according to Baker’s classification, and use of lipofilling. In the group of complications requiring surgical intervention, we recorded the need for revision surgery involving a change of implant model. When estimating the rate of implant substitution due to an incorrect implant choice, we excluded those cases of revision surgery done 5 years after a satisfactory outcome or done for other medical reasons (e.g., change in body weight or shape of the contralateral breast, or a new breast cancer).

### Statistical analyses

To identify clinical factors that may lead to a perioperative change in the selection of a breast implant, we used a multivariable logistic regression model. The model was adjusted for BMI (as a continuous variable), preoperative planning (complete vs partial markings), and type of contralateral breast procedure (mastopexy, reduction mammaplasty, or augmentation vs no procedure). The results were reported as odds ratios and 95% confidence intervals.

A multivariable logistic regression model was also fitted to assess the impact of breast shape (width, height, and projection) on the likelihood of a change of implant choice. For the calculation of odds ratios, the median preoperative measurements were used as reference points. To take into account possible non-linear effects, restricted cubic splines with three knots were used [13]. All statistical analyses were done using R3.02 for Windows, with the rms package added.

## Results

A total of 496 women who underwent unilateral breast reconstruction during the study period (Table 1) were included in this retrospective analysis. About one quarter of the women had unilateral breast reconstruction alone, while the remaining patients had a contralateral procedure for symmetry, with augmentation being more frequent than either mastopexy or reduction mammaplasty. A total of 308 implant choices (62.1%) were changed perioperatively.

**Table 1:** Clinical and surgical data regarding 496 women who had unilateral mastectomy and two-stage breast reconstruction

Variable	Value
Body mass index, mean (SD)	23.6 (3.7)
Preoperative markings, *n* (%)	
Complete	259 (52.2)
Partial	237 (47.8)
Contralateral breast procedure, *n* (%)	
None	127 (25.6)
Mastopexy	100 (20.2)
Reduction mammaplasty	110 (22.2)
Augmentation	159 (32.0)
Change in implant selection, *n* (%)	308 (62.1)

Among the implants selected preoperatively and those actually implanted (Table 2), the majority were from the Natrelle 410 series, which has many more models than the 510 series. The implants with medium or full-height and extra-full projection (MX and FX) were more frequently chosen in both the preoperative and perioperative settings. In our experience, these models are better able to restore the breast profile that is usually flat after mastectomy and tissue atrophy, especially for medium-sized and large breasts. In particular, the most used 410 models were MX550 and LX625 in large breasts; MX445 and MX410 in medium-sized breasts; and MX325, FF290, and MF255 in small breasts. In four cases, a round implant (110 or 120) was chosen in the perioperative setting, for better matching with the contralateral breast and rebuilding of a lower pole overhanging the IMF according to the reconstructive technique used in our institute [14].

**Table 2:** Breast implants selected during preoperative planning and actually implanted during breast reconstruction in 496 women

Implant model	Preoperatively chosen model		Implanted model	
	Cases, *n*	Weight range, g	Cases, *n*	Weight range, g
410 series				
LL	3	135–240	6	135–240
LM	10	190–320	13	190–320
LF	41	240–540	44	205–595
LX	37	290–625	43	255–685
ML	0	–	0	–
MM	11	245–400	21	240–450
MF	65	225–580	65	225–640
MX	197	225–685	166	225–685
FL	1	250	2	250
FM	4	270–670	1	310
FF	12	255–535	18	220–740
FX	101	280–690	88	315–775
510 series				
LX	3	425	2	330–365
MX	11	335–550	19	335–490
FX	0	–	4	310–495
110	0	–	3	150–510
120	0	–	1	550

### Factors associated with a change in implant choice

Multivariable logistic regression was used to identify clinical factors associated with a perioperative change of the implant model selected preoperatively (Table 3). The only variable associated with a change in implant selection was the kind of surgery. In particular, reconstruction with contralateral reduction showed an odds ratio of 1.92, i.e., the likelihood that surgeons changed their plan was nearly twice as high as in reconstruction alone. Similar results were obtained for patients undergoing reconstruction and mastopexy (OR = 2.26). Other factors were not significantly associated with a change of implant selection.

**Table 3:** Results of multivariable logistic regression to test the influence of clinical parameters on the likelihood of a perioperative change in breast implant selection

Factor	Odds ratio (95% CI)	*P* value
BMI (4 kg/m^2^ increment)	1.04 (0.82–1.32)	0.74
Preoperative markings (complete vs partial)	0.87 (0.60–1.26)	0.47
Contralateral surgery		0.01
Mastopexy	2.26 (1.29–3.96)	
Reduction mammaplasty	1.92 (1.12–3.29)	
Augmentation	1.23 (0.75–2.00)	

A second analysis took into consideration the variations in dimensions and weight of the implants between those chosen preoperatively and those actually implanted (Table 4). The median values of height and projection did not change, whereas only small changes in median width and weight were recorded. More than one variable changed in 308 cases (62.1%); width was less frequently changed (53.4%) while height changed the most (67.7%). Width changed more than 0.5 cm (the typical difference in size between consecutive models of implants) in 70 cases (14.1%), while height changed more than 0.5 cm in 215 cases (43.3%).

**Table 4:** Dimensions and weight of breast implants, according to preoperative plan and as actually implanted, for 496 women

Variable	Preoperative choice, median (range)	Implanted model, median (range)	Cases that changed, *n* (%)	Difference, median (range)
Width, cm	13.0 (10.5–16.0)	13.5 (9.7–15.5)	265 (53.4)	0 (−4–2.5)
Height, cm	12.5 (8.6–16. 0)	12.5 (8.6–16.0)	336 (67.7)	0 (−2.9–2.9)
Projection, cm	6.0 (3.0–7.2)	6.0 (2.5–7.1)	297 (59.9)	0 (−3.0–2.7)
Weight, g	410 (135–690)	420 (135–775)	303 (61.1)	0 (−225–240)

**Fig. 2:**
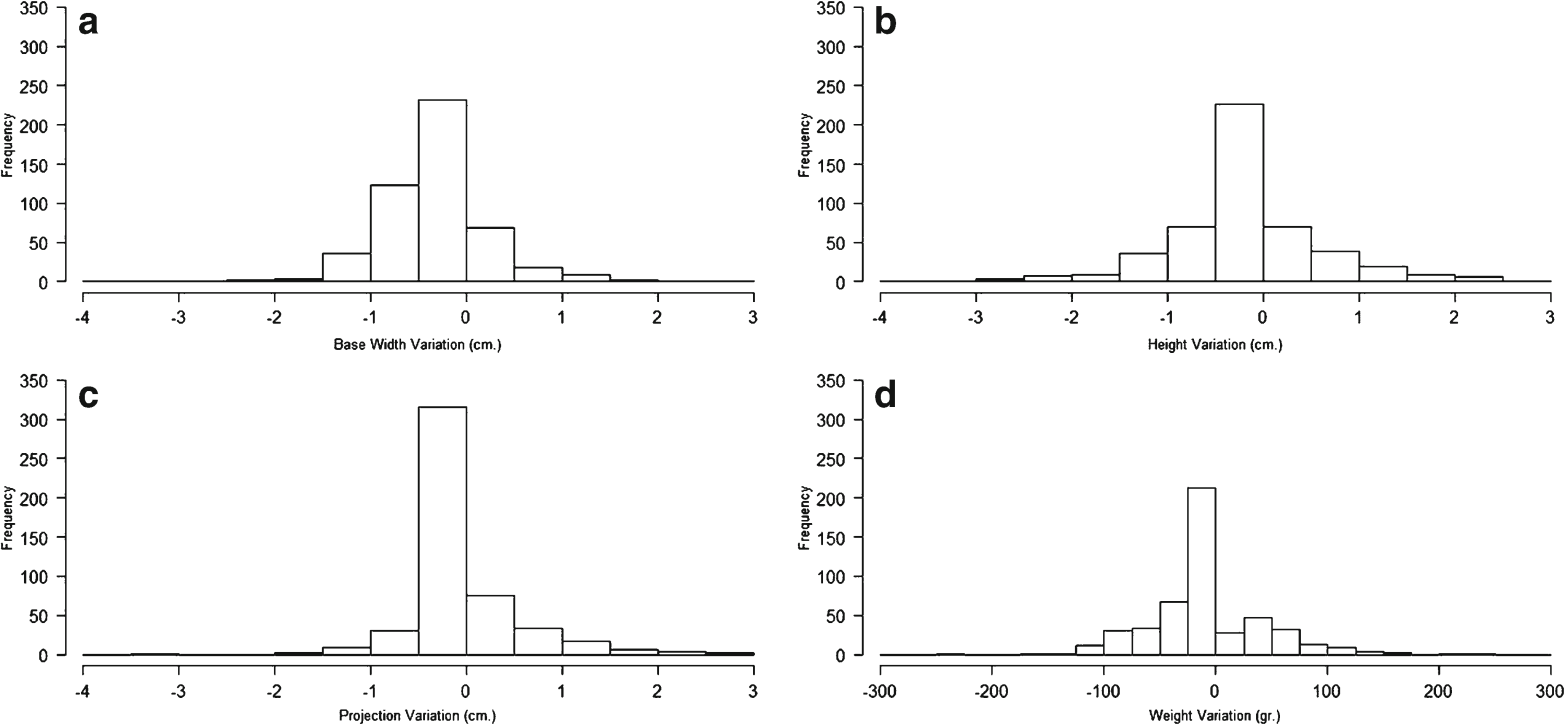
Histograms of the variations in breast implant dimensions between the preoperative plan and the implant actually used. **a** Width. **b** Height. **c** Projection. **d** Weight

Histograms were plotted to illustrate the range of changes in dimensions and weight (Fig. 2). Regarding the differences in dimensions, the interquartile range (IQR), i.e., the range of values that includes the central 50% of cases, was 0.5 cm (−0.5 to 0 cm), 0.8 cm (−0.5 to 0.3 cm), and 0.2 cm (−0.1 to 0.1 cm) for width, height, and projection, respectively, while it was 50 g (−35 to 15 g) for weight. For width, the IQR corresponded to a change (0.5 cm) from one to the next size in the catalog series, while for weight, the IQR (50 g) included two or three models for every category of implant. The graphs show that the highest bar is in the “−0.5 to 0 cm” bin, where the cases with no implant change were placed. For the linear dimensions (Fig. 2a–c), changes of 1 cm or more were rare. For weight (Fig. 2d), the graph illustrates that a few cases required changes beyond 100 g in either direction.

**Fig. 3:**
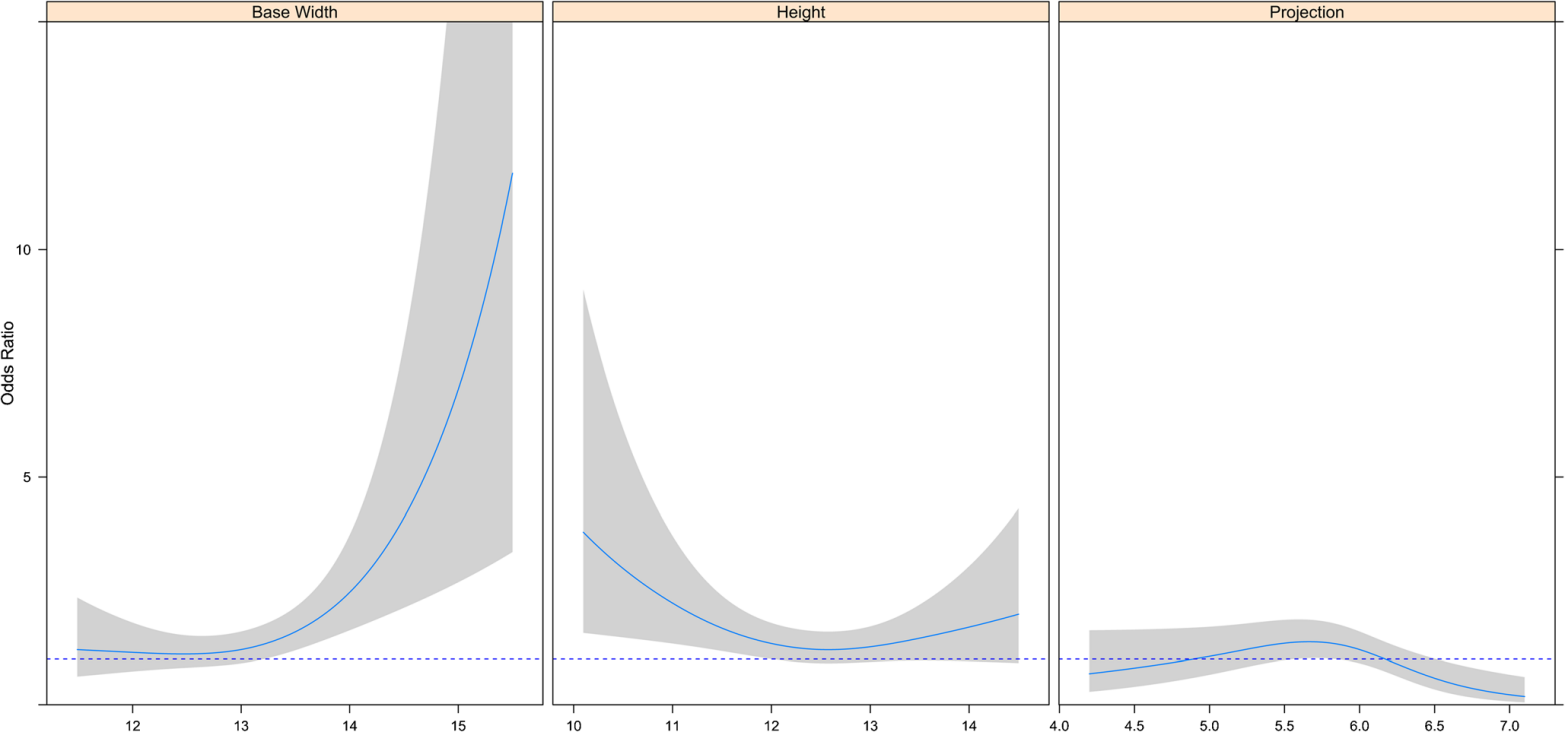
Relationship between preoperative breast dimensions and the odds ratio of a change of implant selection. The *dashed line* corresponds to no effect of the predictor; *shaded bands* are pointwise 0.95 confidence limits for predicted odds ratio

Multivariable logistic regression was used to determine the impact of breast dimensions on the likelihood of a perioperative change in implant selection. For this analysis, the median value of each preoperative measurement as reported in Table 4 was used as the reference value. The trends in odds ratios for each dimension are illustrated in Fig. 3 and summarized with a few examples in Table 5. For preoperative width, the odds ratio for a change in implant was near one for preoperative measurements <13 cm, but for larger widths, it increased precipitously (Fig. 3a). These data suggest that there is a high likelihood of changing the chosen implant when the preoperatively measured width is large. Regarding preoperative height measurements, the odds ratio describing the likelihood of change was near one (suggesting no perioperative change) for intermediate values, while it was substantially above one for larger and above two for smaller preoperative measurements (Fig. 3b). Finally, in preoperative projection measurements, the odds ratio curve deviated from one towards lower values with projection values above 6.5 cm; these results suggest that a change in implant choice is less likely when the projection value is large.

**Table 5:** Results of multivariable logistic regression in shape measurements

Dimension	Odds ratio (95% CI)	*P* value
Width		0.0025
11.5 vs 13.0	0.99 (0.44–2.21)	
14.0 vs 13.0	2.06 (1.25–3.39)	
15.5 vs 13.0	9.66 (1.94–48.2)	
Height		0.0420
10.5 vs 12.5	2.97 (1.47–6.00)	
13.0 vs 12.5	1.27 (0.94–1.72)	
14.5 vs 12.5	1.99 (0.91–4.31)	
Projection		0.0050
4.2 vs 6.0	0.68 (0.28–1.63)	
5.6 vs 6.0	0.44 (0.18–1.07)	
7.1 vs 6.0	0.18 (0.05–0.61)	

### Follow-up

Follow-up data were available for 459 patients (92.5%). The mean follow-up at the time of this study was 16.6 months (range, 4–124 months). Radiotherapy had been administered in 37 cases. The oncological follow-up recorded four local recurrences, eight contralateral cancers, and ten distant metastases. Complications requiring a surgical intervention were recorded in 53 patients (11.5%) including six cases in which the implant was affected: four cases of infection or extrusion (requiring implant removal after 2 months) and two cases of skin necrosis treated by latissimus dorsi flaps and the use of a smaller implant (Table 6). The rate of implant substitution (considering only those cases attributable to a poor implant choice and not to other factors) was 5.9%. In particular, 27 patients had their implant changed with a different model (24 operations in our hospital and three in other hospitals) in a median period of 20 months (range, 2 to 50 months). Nine of these patients had Baker grade III or IV capsular contractures. Only one case of implant substitution also had a lipofilling procedure immediately, while four patients underwent delayed lipofilling. The surgeons’ evaluation of esthetic outcome was predominantly good for breast shape (67.1%) and symmetry (60.3%), and 69.9% of patients also reported a good level of satisfaction.

**Table 6:** Follow-up data for 459 patients

Variable	Cases, *n* (%)
Complications (requiring a surgical intervention)	53 (11.5)
Revision surgery for implant change	27 (5.9)
Breast shape	
Good	308 (67.1)
Average	81 (17.6)
Poor	58 (12.6)
Data missing	12 (2.6)
Symmetry	
Good	277 (60.3)
Average	145 (31.6)
Poor	25 (5.4)
Data missing	12 (2.6)
Patient satisfaction	
Good	321 (69.9)
Average	102 (22.2)
Poor	24 (5.2)
Data missing	12 (2.6)
Capsular contracture	
I normal	39 (8.5)
II	330 (71.9)
III	54 (11.7)
IV severe	4 (0.8)
Data missing	32 (6.9)
Remodeling by lipofilling	4 (0.8)

## Discussion

**Fig. 4:**
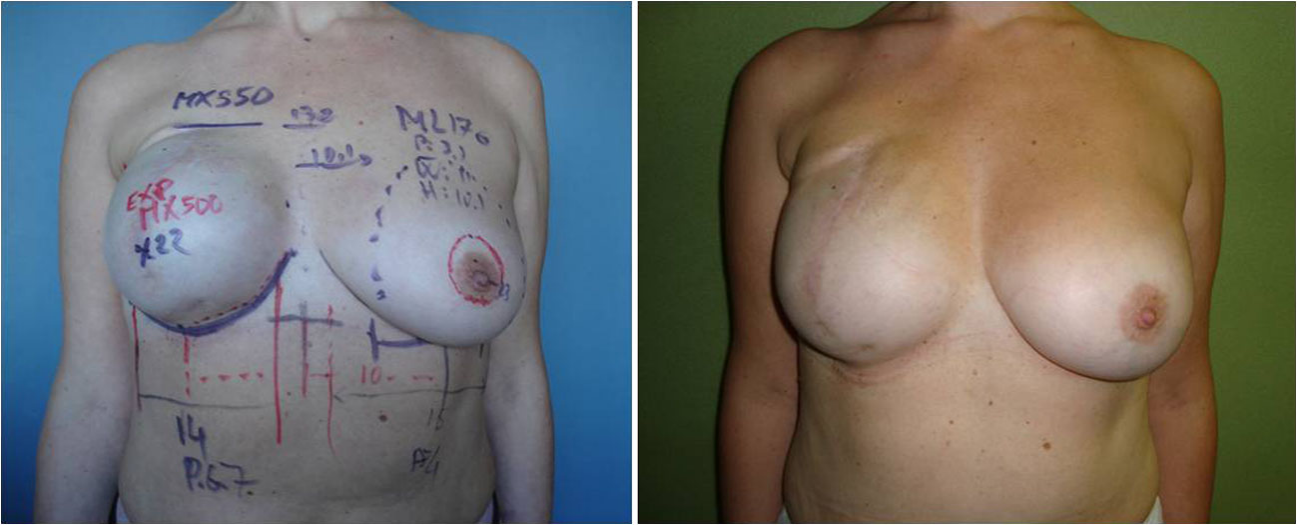
Unilateral expander substitution and contralateral augmentation without mastopexy. The preoperative photograph *(left)* and postoperative photograph at 12 months *(right)* show the planned choices that were selected during surgery (expander 133MX 500 cm^3^ changed with 410MX 550 g and ML 170 g in the contralateral breast)

To our knowledge, this is the first study to provide statistical data on the preoperative selection of implants in two-stage breast reconstruction. Our study shows that about 62% of implant selections made in the preoperative period were changed during surgery. Changes in implant dimensions were generally less than 0.5 cm, requiring the surgeon to select the next implant in the series. Width changes were less frequent than height changes and occurred primarily with small implants. Height changes were the most frequent changes and were the least predictable for the greatest and smallest height measurements. Projection changes accounted for a few millimeters only, in particular with projection values exceeding 6 cm. The likelihood of a change in implant selection was higher when the preoperative breast width was >13 cm or when contralateral surgery was planned, in particular mastopexy (Fig. 4). The considerable influence of mastopexy may be explained by the difficulty in predicting the projection of the lifted breast, with particular attention being paid to upper pole fullness.

This study reveals the importance of accurate measurements of width and height of the breast, with weight having lesser importance. This may entail a shift from round implants, where volume was the optimal measure, to anatomical implants, where shape and size can vary greatly despite similar volume [15]. Width is the focal point of every implant choice for reconstruction [9, 10].

**Fig. 5:**
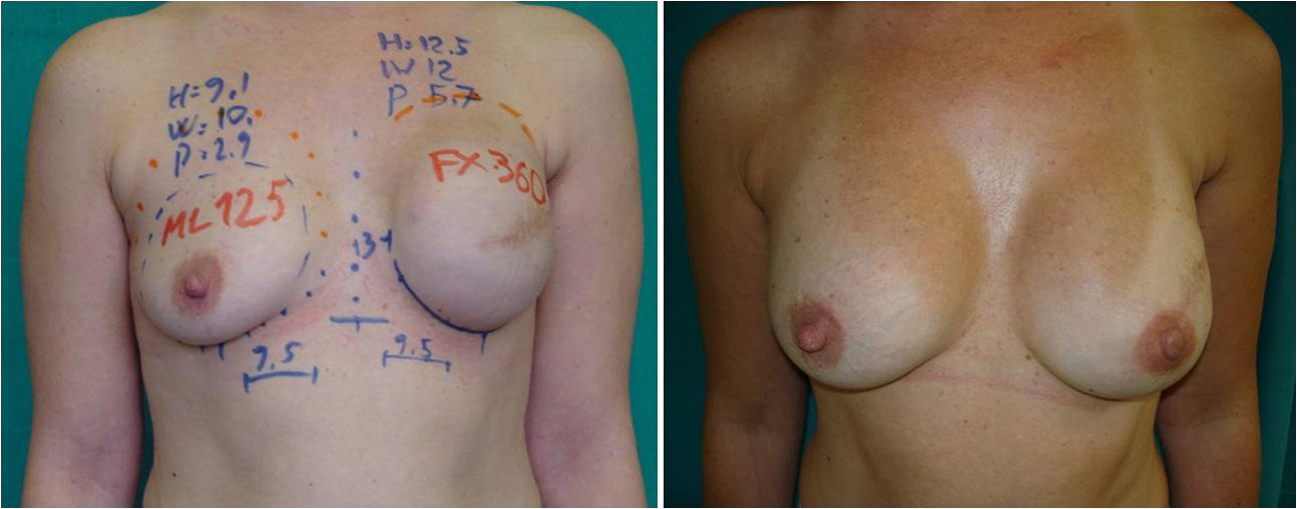
Unilateral expander substitution and contralateral augmentation. The preoperative photograph *(left)* shows the implant selection, which was altered during surgery. On the left side, the 133MV 300 cm^3^ expander was exchanged with 410FX 410 g, and on the right side, a fixed-volume 410FL 190 g was placed. The postoperative photograph demonstrates good symmetry at 24 months *(right)*. The variations in dimensions are as follows: left: width +0.5 cm, height +0.5 cm, and projection +0.3 cm; right: width +1.0 cm, height +2.4 cm, projection +0.2 cm

**Fig. 6:**
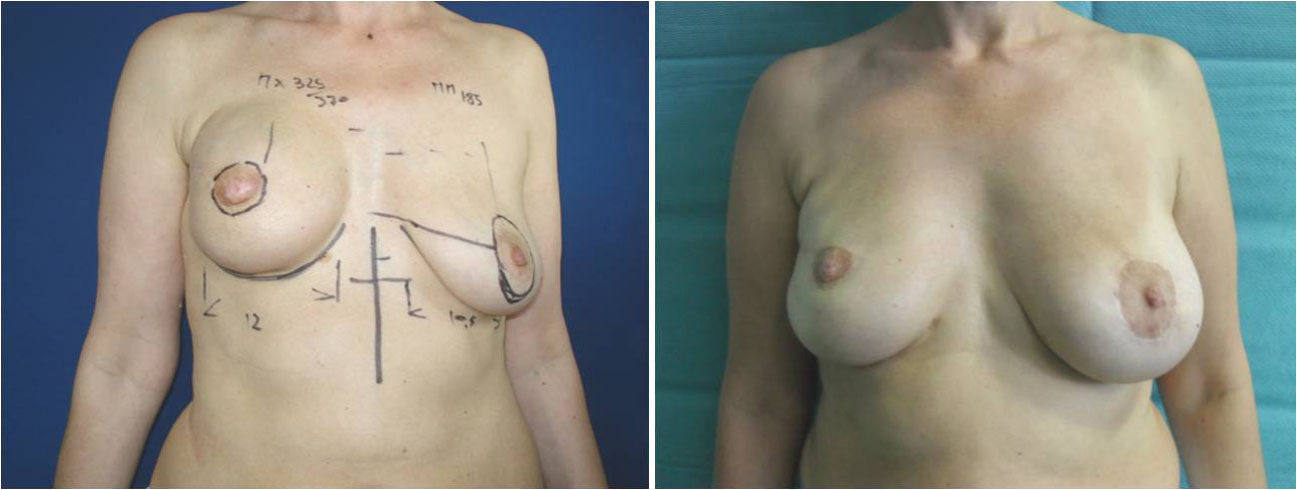
Unilateral expander substitution and contralateral augmentation with round-block mastopexy. The preoperative photograph (*left*) shows the selected implants; the postoperative view was taken at 12 months (*right*). Planning foresaw the use of 410MX 325 g on the right and 410MM 185 g on the left. The reconstructive implant was altered to 410FM 350 g during surgical refinement. Based on the vertical enlargement of the pocket produced by the soft tissue responsiveness and the rebuilding of the inframammary fold, the implant parameters were changed (more height and less projection)

The higher rate of change in implant height may reflect the availability of devices offered by the manufacturer (low, medium, full) for an individual base width; however, more technical reasons should also be considered, such as (1) overexpansion of the upper pole by a temporary expander, which can hide the actual soft tissue thickness and make an accurate pinch test result more difficult to obtain; (2) rib cage deformity after expansion, which may be appreciated once the expander is removed (Fig. 5); (3) IMF level, which must be planned preoperatively but is delineated perioperatively [14, 16]; and (4) grade of ptosis achievable in some cases using the technique of inframammary remodeling (Fig. 6) [17]. This procedure may result in changes in projection and shape to fit the lowermost part of the implant in the hanging envelope of the pocket (less projected anatomical implant and sometimes round implants).

The choice of projection showed the least variation, but this may be partially due to the lower range of options compared with those offered by width and height. Another reason may be the preference for extra-projected models, reducing the range available. It may be that the real variations can be explained either by the effect produced by contralateral surgery or by thoracic flattening due to posterior expansion, which is only assessable during the operation.

The appropriateness of the implant choice at the time of surgery was confirmed by patient satisfaction and minimal requests for surgical replacement of implants. Nevertheless, more effort should be made to improve the predictability of the preoperative plan. We could improve the preoperative plan by measuring medial and lateral thickness more accurately in the non-affected breast (especially when planning a mastopexy) and not only at the mastectomy site. According to the statistical outcomes, we should decrease the preselected implant width by about 0.5 cm when it exceeds 13 cm and mastopexy is planned. Otherwise, when planning a mastopexy or breast reduction, we should always measure the parenchyma thickness of the non-affected breast by the pinch test medially and laterally, with the breast displaced laterally and medially, respectively, so as to simulate medial and lateral pillar advancement during surgery.

The search for a simple predictive formula for implant selection is challenging, especially when multiple variables need to be accounted for. These variables may be identified in the preoperative planning stage and include certain characteristics of the breast (skin envelope, scar tissue, muscle coverage, IMF position, nipple position), as well as size and ptosis of the opposite breast. Sigurdson and Kirkland used multiple linear regression analysis of anthropomorphic measurements to develop a formula to predict breast volume in case of breast hypertrophy [18]. Descamps et al. used multiple regression analysis to predict resection weight for reduction mammoplasty [19].

The majority of decision-making methods for preoperative planning do not come from the results of multivariable logistic regression models or, more generally, clinical studies. A recent systematic review of implant size selection systems for breast augmentation reports that only 12% of articles included outcomes that could be compared to accepted literature values or industry standards [20]. The present study has critically addressed this problem for breast reconstruction and searched for mathematical intervals of confidence.

Chae et al. published a literature review concerning volumetric analysis for esthetic planning in breast reconstruction spanning from 1950 to 2015 [21]. They argued that thermoplastic casting, direct anthropomorphic measurement, 2D imaging, and computed tomography/magnetic resonance imaging scans have mostly been unreliable, difficult to execute, and characterized by limited practicability. The only exception is 3D surface imaging, even if limited by high costs and the lack of a high level of evidence.

Breast implant surgery has been based on anthropometric parameters and implant width, height, and projection, but we are aware that no fully reliable methods are available in case of asymmetrical breast surgery. The present study has tried to quantify the risk of pitfalls. It shows that preoperative planning in breast reconstruction is still difficult and that implant selection can be reevaluated intraoperatively when major refinements of the pocket or contralateral surgery are being planned.

Volume/weight measurement is an important aspect. We have analyzed the volume/weight differences in our study, but we believe that volume information cannot be a selective parameter for choosing an anatomical implant, especially in breast reconstruction. Volume information is useful for the patient’s comprehension of our planning; it is also still useful for avoiding major sizing errors in case of breast asymmetry.

The study confirms that width is of great importance in the anthropometric methods of implant planning because it is the factor with the least likelihood of change, while weight has lesser importance. The height of the selected implant is at the highest risk of being changed during surgery.

Although it has not been possible to statistically validate a safe algorithm for implant selection in the preoperative setting based on three dimensions, the results show that preoperative measurements (in particular width) are significantly more reliable in smaller implants (<335 g). Moreover, preoperative selection of implant width can become reliable with a tolerable margin of ±0.5 cm. Width should serve as a starting point for implant selection and may vary, requiring the availability of implant sizes above and below the selected implant.

Surgeons should be aware of the lesser predictability of implant selection in the case of contralateral surgery, in particular mastopexy, and when large implants are used. We recommend that clinics involved in breast reconstruction be equipped with a large stock of implants in various shapes and sizes.

In conclusion, the multivariable logistic regression model demonstrates that preselection of implants with anthropometric evaluation alone in the case of contralateral mastopexy, breast reduction, or augmention is rather difficult, with potential risks and a possible need for further intraoperative selection. Intraoperative recalculation of breast dimensions by ruler and ultimately the use of sizers is required. This dual planning selection system may have an advantage in terms of patient reoperation rates.

In the future, novel advanced methods of breast volumetric analysis, such as web-based 3D surface imaging programs, 4D imaging, and 3D printing, can help surgeons perform reliable preoperative planning, thanks to their potential advantages: efficiency, easy and fast application, and low cost.
